# How to Improve Compliance with Protective Health Measures during the COVID-19 Outbreak: Testing a Moderated Mediation Model and Machine Learning Algorithms

**DOI:** 10.3390/ijerph17197252

**Published:** 2020-10-04

**Authors:** Paolo Roma, Merylin Monaro, Laura Muzi, Marco Colasanti, Eleonora Ricci, Silvia Biondi, Christian Napoli, Stefano Ferracuti, Cristina Mazza

**Affiliations:** 1 Department of Human Neuroscience, Sapienza University of Rome, 00185 Rome, Italy; paolo.roma@uniroma1.it (P.R.); marco.colasanti@hotmail.com (M.C.); eleonoraricci25@gmail.com (E.R.); silviabiondi14@gmail.com (S.B.); stefano.ferracuti@uniroma1.it (S.F.); 2 Department of General Psychology, University of Padova, 35131 Padova, Italy; merylin.monaro@unipd.it; 3 Department of Dynamic and Clinical Psychology, Sapienza University of Rome, 00185 Rome, Italy; laura1.muzi@uniroma1.it; 4 Department of Medical Surgical Science and Translational Medicine, Sapienza University of Rome, 00189 Rome, Italy; christian.napoli@uniroma1.it; 5 Department of Neuroscience, Imaging and Clinical Sciences, University “G.d’Annunzio”, 66100 Chieti-Pescara, Italy

**Keywords:** COVID-19, compliance, efficacy, risk perception, civic engagement, personality

## Abstract

In the wake of the sudden spread of COVID-19, a large amount of the Italian population practiced incongruous behaviors with the protective health measures. The present study aimed at examining psychological and psychosocial variables that could predict behavioral compliance. An online survey was administered from 18–22 March 2020 to 2766 participants. Paired sample *t*-tests were run to compare efficacy perception with behavioral compliance. Mediation and moderated mediation models were constructed to explore the association between perceived efficacy and compliance, mediated by self-efficacy and moderated by risk perception and civic attitudes. Machine learning algorithms were trained to predict which individuals would be more likely to comply with protective measures. Results indicated significantly lower scores in behavioral compliance than efficacy perception. Risk perception and civic attitudes as moderators rendered the mediating effect of self-efficacy insignificant. Perceived efficacy on the adoption of recommended behaviors varied in accordance with risk perception and civic engagement. The 14 collected variables, entered as predictors in machine learning models, produced an ROC area in the range of 0.82–0.91 classifying individuals as high versus low compliance. Overall, these findings could be helpful in guiding age-tailored information/advertising campaigns in countries affected by COVID-19 and directing further research on behavioral compliance.

## 1. Introduction

Public willingness to comply with the protective health measures proposed by authorities is critical for controlling the outcomes of an infectious disease outbreak [1], given that “behavioral changes can significantly affect the epidemic spread both qualitatively […] and quantitatively” [2]. Coronavirus disease 2019 (COVID-19, also known as 2019-nCoV), an acute respiratory illness with an unknown cause, emerged in China in December 2019 and, since then, has spread rapidly throughout most of the world. In January 2020, the World Health Organization declared it an international public health emergency and, shortly thereafter, a global pandemic [3]. On 30 January 2020, the first two cases of COVID-19 in Italy were confirmed by the Italian government. In the following weeks and months, the health emergency generated devastating consequences for both local residents and national health workers. Due to the lack of supported treatments and vaccines, Italy (similar to other countries) implemented a policy of social and physical distancing, as well as a mandatory recommendation to “stay at home”. However, the virus continued to escalate at an overwhelming rate, due in part to the failure of a portion of Italian residents to observe the recommended health measures, despite the government’s prevention information campaigns (issued from February onwards). To dissuade unsafe behaviors, the government was forced, on 19 March, to introduce penalties for those violating the guidance by leaving their domiciles for reasons other than stringent and absolute need. It is worth noting that, at this point in time, the contagion was already very advanced: on 18 March, the official cases reported by the government included 33,190 infected, 4440 recovered, and 3400 deceased [4,5,6,7]. 

The present research stemmed from the observation that, throughout this health crisis, Italian residents (similar to those in other affected countries) have engaged in incongruous behaviors, thereby severely limiting the effective management of COVID-19. To investigate this phenomenon more deeply, we selected and explored the effect of multiple psychological and psychosocial variables that were thought to relate to individual differences in compliance with the intervention measures and safety behaviors during the first phases of the COVID-19 outbreak in Italy.

### 1.1. Perceived Efficacy and Compliance

The belief that a recommended health behavior will have positive consequences and/or will reduce the public health threat (or its seriousness) is commonly defined as perceived efficacy or perceived benefits. In actuality, these terms indicate slightly different constructs, originating from different theoretical frameworks: the health belief model (HBM) [8] and protection motivation theory (PMT) [9,10], respectively. Although perceived benefits also include non-health-related positive outcomes (e.g., having more money after quitting smoking), the term is often used synonymously with perceived efficacy. In this study, we use the term perceived efficacy very specifically to describe people’s perception of the efficacy of the recommended preventive measures in reducing the risk of contagion. Perceived efficacy has been studied in relation to a variety of general health measures, including engaging in physical activity, receiving vaccinations, and complying with medical treatment among psychiatric outpatients [11,12,13]; the literature suggests that it is a key determinant for compliance with preventive health behaviors and that “only when a person feels that the recommended behavior is likely to lead to the desired outcome will adoption of the recommendations occur” ([14], p. 193). Perceived efficacy has also been studied in the context of past epidemics/pandemics (e.g., H1N1) and the current COVID-19 outbreak, demonstrating that it is one of the strongest predictors of compliance with preventive health behaviors [15,16,17].

### 1.2. The Mediating Role of Self-Efficacy 

Self-efficacy is defined as “people’s beliefs about their capabilities to produce designated levels of performance that exercise influence over events that affect their lives” ([18], p. 71). Of note, the concept also refers to an individual’s belief about their ability to perform specific behaviors in particular situations [19]. A direct relationship between self-efficacy and behavioral change has been found in many health contexts (relating to e.g., cigarette smoking, weight control, contraceptive behavior, and alcohol abuse) [20,21] and during past pandemics [22], indicating a strong relationship between self-efficacy and health behavior change and maintenance. According to the health action process approach (HAPA) [23], three specific cognitions lead to an intention to act (i.e., motivation): (a) perceiving oneself as at risk (i.e., a risk perception), (b) believing that the recommended health behavior will reduce the threat (i.e., an efficacy perception), and (c) self-efficacy. Although efficacy perception has been found to be a stronger predictor than self-efficacy in developing the intention to act [24], “individuals need to know the contingencies between behaviors and outcomes […], but they also need to be confident that they can really perform the behavior in question” (p. 493). Accordingly, the present study aimed at revealing whether self-efficacy plays a mediating role in this relationship. We posited that feeling able to perform the recommended health behaviors would mediate the relationship between the perceived efficacy of those measures and compliance.

Research Question 1 (RQ1): Self-efficacy would mediate the relationship between the perceived efficacy of the government health measures and compliance (simple mediational model). Higher levels of perceived efficacy would increase compliance through higher self-efficacy. 

### 1.3. The Moderating Role of Risk Perception

Risk perception, defined as an individual’s belief about the risk of potential harm, is a key construct of many theoretical frameworks for health behaviors (e.g., HBM, PMT, HAPA). Risk perception is influenced by the perceived severity of the specific health threat and the perceived likelihood of harm. As noted by Brewer et al. [25], previous studies have often used the terms “likelihood” and “vulnerability”/“susceptibility” interchangeably, even though the first represents “one’s probability of being harmed by a hazard under certain behavior conditions” (p. 136), whereas the second can be defined as individual resistance or constitutional vulnerability.

Research on past epidemics/pandemics (e.g., H1N1) has not only shown risk perception to be a key driver of health behaviors, but it has also consistently found an association between risk perception and precautionary behaviors [26,27,28], even though a meta-analysis highlighted that this association involves only small effect sizes [25], especially with respect to perceived severity (*r* = 0.16). In the context of health psychology, significant interactions between risk perception and self-efficacy have been found; for example, research has identified an association between motivation to think about cardiovascular disease, use of health information, and knowledge acquisition [29]. However, some research has reported no significant direct effect of risk perception on preparedness measures. Bourque et al. [30] for instance, found that the effect of risk perception was largely mediated by knowledge, perceived efficacy, and milling behavior in household preparedness for terrorism behavior in the United States.

Research Question 2 (RQ2): Risk perception would moderate the relationship between the perceived efficacy of the government health measures and compliance (Research Question 2a), as well as the positive relationship between the perceived efficacy of the health measures and self-efficacy (Research Question 2b), and the positive relationship between self-efficacy and compliance (Research Question 2c) (moderated mediation models). Higher perceived efficacy of the health measures would be associated with increased compliance with protective health measures, especially alongside higher risk perception. 

### 1.4. The Moderating Role of Civic Engagement

Civic engagement can be defined as “the process of believing that one can and should make a difference in enhancing his or her community” [31]. It is typically thought to have two dimensions: attitudes and behaviors. However, since the present study was focused on cognitive variables, we primarily examined civic attitudes. 

Due to the lack of a strong theoretical model for civic engagement in health contexts, no previous research has examined the link between civic engagement and health behaviors (or specifically, compliance with health measures). However, studies have focused on specific aspects of civic engagement, such as perceived moral responsibility. For example, a recent study [13] investigating the vaccination rate among nurses in Hong Kong considering HBM constructs and using perceived moral responsibility as a moderator found an insignificant effect. Furthermore, previous studies have examined individuals’ level of information regarding community health concerns during pandemics (including the current COVID-19 pandemic), demonstrating a positive association between knowledge about a specific threat and preventive behaviors [16,32,33]. These results suggest that promoting knowledge about COVID-19 might encourage the adoption of preventive behaviors. 

Other facets of civic engagement—namely political efficacy and an interest in public affairs—have been found to be positively associated with self-efficacy [34]. Furthermore, research on mobile donation as a new platform for technology-mediated civic engagement has demonstrated that perceived effectiveness (i.e., the degree to which consumers believe that the company will really donate as much as promised and that this donation will actually reach the needy recipients) has a positive effect on the intention to donate via mobile phone [35].

In this vein, the present study sought to explore the relationship between civic engagement (specifically, civic attitudes), perceived efficacy, self-efficacy, and compliance with protective health measures during the COVID-19 pandemic. The COVID-19 outbreak presents a unique opportunity to investigate the role of civic engagement—together with the aforementioned variables—since even asymptomatic individuals are able to spread the virus to vulnerable populations (e.g., older adults and the elderly) [36]. Therefore, we included this variable as a moderator because it could potentially provide insight into how to improve compliance to the recommended preventive behaviors. Indeed, in the Research Question 3 (RQ3): Civic attitudes would moderate the relationship between the perceived efficacy of the government health measures and compliance (Research Question 3a), as well as the positive relationship between the perceived efficacy of the health measures and self-efficacy (Research Question 3b), and the positive relationship between self-efficacy and compliance (Research Question 3c) (moderated mediation models). Higher perceived efficacy of the health measures would be associated with increased compliance with the protective health measures, especially alongside higher levels of civic attitudes. 

**Figure 1:**
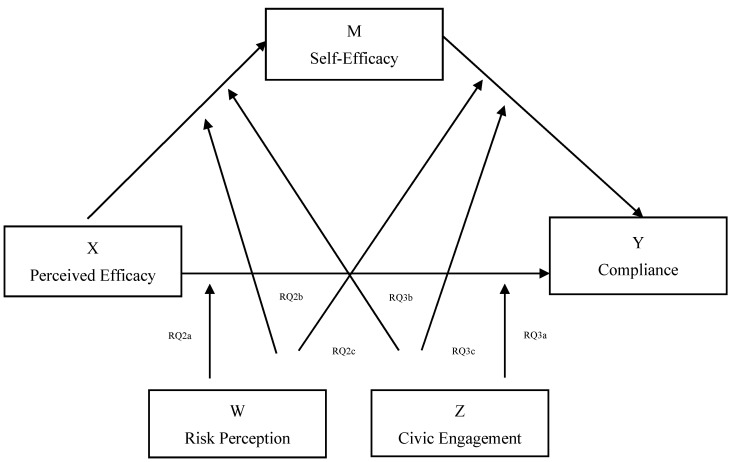
Proposed moderated mediation model.

Figure 1 shows the proposed moderated mediation model.

### 1.5. The Role of Age, Education, and Personality Dysfunction

The results of a previous review on demographic and attitudinal determinants of compliance with protective measures during a pandemic showed that being older, female, more educated, and non-White were associated with a higher likelihood of adopting the recommended health behaviors [37]. More recently, several studies have investigated which factors were associated with compliance with the preventive measures during the COVID-19 pandemic. The results indicated that the demographic variables of male gender and younger age were associated with lower levels of compliance [38,39,40], while—albeit not consistently—the demographic variables of a higher level of education and being married were associated with greater compliance [38]. 

Together with age and education, we included level of personality dysfunction (as assessed by the DSM-5 approach [41]) as a control variable, as several studies have shown that people react differently to threats according to certain personality traits [42,43]. For instance, individuals with high levels of antagonism, which refers to aggressive tendencies accompanied by assertions of dominance and grandiosity, may argue with other people when their desires are not satisfied [44]. Moreover, high levels of disinhibition are commonly related to greater impulsivity and sensation seeking, which could lead to a tendency to ignore real dangers or threats. Finally, among personality traits, only agreeableness was associated with greater compliance, whereas aspects of the dark triad (i.e., narcissism, Machiavellianism, and psychopathy) and antisocial traits were predictive of lower levels of compliance [45,46]. Despite the paucity of studies on personality variables and compliance with government recommendations during past pandemics, it is possible that higher levels of personality dysfunction could interfere with behavioral compliance. 

### 1.6. Predictive Models

Recently, researchers from a range of scientific fields (including the clinical and social sciences) have begun to emphasize the increased need to focus on prediction, rather than explanation, during data analysis [47]. Machine learning (ML) is a branch of artificial intelligence that focuses on data prediction. ML algorithms automatically learn information from a set of data and make predictions on unseen data without being explicitly programmed to do so. ML techniques have been shown to be particularly useful in predicting human behavior, including high-risk behavior [48,49,50,51]. Indeed, one of the main advantages of ML is that it enables inferences to be made at the individual level, whereas traditional statistical methods focus primarily on the group level [52]. Thus, ML predictive models support researchers in making predictions for individual subjects, which is particularly useful for the development of personalized and targeted prevention campaigns. In the present study, ML algorithms were applied to the 14 psychosocial variables to predict individuals’ likelihood of complying with the COVID-19 protective measures. 

Research Question 4 (RQ4): Which variables would predict, with maximal accuracy, high versus low compliance with the protective health measures prescribed by the Italian government? 

## 2. Materials and Methods

### 2.1. Procedures

The research team assembled an online survey inspired by the literature and collected data over five days (18–22 March 2020). The questionnaire was administered cross-sectionally on an online survey platform, which participants accessed via a designated link. The link was disseminated through the main means of communication and social networks, in order to reach a large number of subjects. The study was approved by the local ethics committee (Board of the Department of Human Neuroscience, Faculty of Medicine and Dentistry, Sapienza University of Rome).

### 2.2. Measures

#### 2.2.1. Behavioral Compliance 

Ten questions were designed to investigate compliance with the COVID-19 protective measures (e.g., “It is suggested that all persons avoid crowded places. Are you complying with this?”). These questions were assessed on a five-point Likert scale ranging from 1 (slightly) to 5 (extremely), with Cronbach’s alpha of 0.84. 

#### 2.2.2. Perceived Efficacy

When COVID-19 became pervasive, the Italian government announced guidelines for preventing infection, including “Disinfect hands often” and “Stay at home”. Accordingly, 10 statements were provided to measure the perceived efficacy of 10 protective guidelines (e.g., “It is suggested that all persons avoid crowded places. Do you find this useful?”) using a five-point Likert scale ranging from 1 (hardly) to 5 (extremely). Cronbach’s alpha was 0.93.

#### 2.2.3. Self-Efficacy

Participants’ self-efficacy with respect to protecting themselves from COVID-19 was measured using three questions (e.g., “I am confident in my ability to protect myself from COVID-19”) adapted from previously validated measures [22], which were assessed on a five-point Likert scale ranging from 1 (strongly disagree) to 5 (strongly agree). Cronbach’s alpha was an acceptable 0.62.

#### 2.2.4. Risk Perception

Risk perception was assessed through two variables, perceived severity and perceived likelihood, using items adapted from Cho and Lee [22] and Liao, Cowling, Lam, Ng, and Fielding [53]. Perceived severity was assessed using four items (e.g., “If I got COVID-19, it would be severe”), with a Cronbach’s alpha of 0.70. Perceived likelihood was assessed using two items (“How likely is it that you will get COVID-19 in this period?”), with Cronbach’s alpha of 0.80. Six questions were assessed on a five-point Likert scale ranging from 1 (not likely at all) to 5 (certain). 

#### 2.2.5. Civic Engagement 

The Civic Engagement Scale (CES) [31] was employed. The CES is a 14-item scale based on an understanding of civic engagement as “the process of believing that one can and should make a difference in enhancing his or her community”. It measures two specific aspects of civic engagement: attitudes and behaviors. The first subscale (Attitudes), composed of eight items, assesses civic attitudes in terms of “the personal beliefs and feelings that individuals have about their own involvement in their community and their perceived ability to make a difference in that community”. The second subscale (Behaviors), composed of six items, assesses civic behaviors in terms of “the actions that people take to actively attempt to engage and make a difference in their community”. Both the Attitudes and the Behaviors subscale obtained high reliability in the validation study, with Cronbach’s alphas of 0.91 and 0.85, respectively. In the present sample, Cronbach’s alpha was 0.87 for both scales, and each item was assessed on a five-point Likert scale ranging from 1 (strongly disagree) to 5 (strongly agree). 

#### 2.2.6. Personality Dysfunction

Personality dysfunction was investigated using the Personality Inventory for DSM-5—Brief Form—Adult (PID-5-BF) [54]. The PID-5-BF is a 25-item self-rated personality traits assessment. It measures five personality trait domains: negative affect, detachment, antagonism, disinhibition, and psychoticism. Each domain is measured through five items, which are rated on a four-point Likert scale ranging from 0 (very false or often false) to 3 (very true or often true). The overall measure generates scores in the range 0–75, with higher scores indicating greater overall personality dysfunction. Each trait domain receives a score in the range 0–5, with higher scores indicating greater dysfunction in that specific personality trait domain.

### 2.3. Participants

A total of 2812 respondents participated in the survey. All participants were aged 18 years or older and were living in Italy. The online survey was closed on the sixth day following dissemination of the link. All participants voluntarily responded to the anonymous survey and indicated their informed consent within the survey. The procedures were clearly explained, and participants could interrupt or quit the survey at any point without explaining their reasons for doing so. Two respondents were excluded from the sample because they were younger than 18 years, and 44 participants were excluded because they lived outside of Italy during the outbreak. 

The final sample consisted of 2766 participants: 1982 (71.7%) females and 784 (28.3%) males. The mean age of the sample was 32.94 (13.2; range 18–90 years), and the majority was Italian citizens (*N* = 2739, 99%). Most of the sample (*N* = 1194, 43.2%) held a high school degree and were unmarried (*N* = 1866, 67.5%), unemployed (*N* = 1165, 42.1%), and childless (*N* = 2130, 77%). Furthermore, most participants reported to be staying at home (*N* = 2368, 85.6%) and going out up to once per day (*N* = 2559, 92.5%). 

## 3. Statistical Analysis 

A paired sample *t*-test was used to compare efficacy perception with behavioral compliance. Cohen’s *d* [55] effect size was inspected for each significant effect. 

The mediation and moderated mediation models were run using PROCESS version 3.5 [56], as developed by Preacher and Hayes [57] for SPSS, version 25 (IBM, Armonk, NY, USA). Moderated mediation test simple mediation models (i.e., determining whether a given variable or mediator accounts for some or all of the relationship between two other variables) that may differ according to a further variable (e.g., if the mediation pathway is only present for individuals with higher or lower levels of certain variables). PROCESS estimates indirect effects (i.e., mediation) and conditional indirect effects (i.e., moderated mediation) using bootstrap confidence intervals. In the present study, the bias-corrected 95% confidence interval (CI) was calculated using 5000 bootstrapping resamples. Effects were considered significant if the resulting CI did not contain 0. All measures, including compliance with protective health measures, were treated as continuous variables except for the ordinal covariate “education”. Considering PROCESS model templates [58], we first tested a simple mediation model (Model 4) to explore if the association between perceived efficacy of the recommended health measures and compliance was mediated by self-efficacy. Next, we tested Model 76 to verify the moderated effect of risk perception and civic attitudes on the direct and indirect effects of the perceived efficacy of the health measures on compliance. “High” and “low” levels of both moderators were determined at one standard deviation above and below the mean of each scale. Taking into account previous findings [38,39,40,42,43], all analyses controlled for age, education, and level of personality dysfunction. Finally, ML models were trained and tested in WEKA 3.9 [59]. The procedure used to build the models is reported in the “Results” section.

## 4. Results

Descriptive statistics and scale correlations are reported in Table 1.

### 4.1. Paired Sample *t*-Test 

A paired sample *t*-test was conducted to investigate differences between the perceived efficacy of the recommended safety behaviors and compliance. The result was statistically significant—*t* (2765) = 37.384; *p* < 0.001—with an effect size (= 0.711) approaching Cohen’s [55] standard for a large effect (= 0.80). The results further indicated a statistically significant reduction in scores for behavioral compliance (M = 41.7; SD = 6.20) relative to perceived efficacy (M = 44.8; SD = 6.17). Subsequent paired sample *t*-tests were run for each safety measure prescribed by the Italian government (e.g., “Avoid hugs”, “Avoid handshakes,” etc.). Since the category of protective health measures was very heterogeneous, being composed of 10 behavioral types, we conducted *t*-tests for each measure. Our aim was to thoroughly investigate whether a difference between perceived efficacy and compliance impacted some behaviors more than others. *T*-test results were significant for all protective measures except for “Avoid handshakes”: *t* (2765) = 1.253; *p* < 0.210; *d* = 0.024 (see Table 2). 

### 4.2. Mediation Model

Research Question 1 postulated that self-efficacy would mediate the relationship between the perceived efficacy of the recommended health measures and compliance. As shown in Table 3, the total effect of perceived efficacy on compliance was significant (*B* = 0.750 (SE = 0.01); *p* < 0.001 (CI = 0.725, 0.775)). The mediation analyses showed that the indirect effect of perceived efficacy on compliance via self-efficacy was positive (0.034) and the bootstrapped 95% CI did not include 0 (0.026, 0.044). Furthermore, the covariates age, education, and personality dysfunction were significant: the first showed a positive association with behavioral compliance, whereas the others showed a negative association with the outcome variable. The final mediation model explained 60% of the total variance in compliance with the health measures: 97% of the total effect on compliance was explained by the direct effect of perceived efficacy, whereas 3% was explained by the indirect effect of the mediator.

### 4.3. Moderated Mediation Model

The results of the moderated mediation model related to conditional indirect effects, as presented in Table 4. Research Question 2 was partially confirmed: risk perception emerged as a significant moderator in the relationship between the perceived efficacy of the recommended health measures and compliance (Research Question 2a), whereas no moderation effects of this variable emerged in either the relationship between perceived efficacy and self-efficacy or the relationship between self-efficacy and compliance (Research Questions 2b and 2c). 

Similar results were found for the moderating role of civic attitudes. As shown in Table 4, this variable moderated the direct effect of perceived efficacy on compliance (Research Question 3a). However, no significant effects were found in the relationship between perceived efficacy and self-efficacy or the relationship between self-efficacy and compliance (Research Questions 3b and 3c). 

Further, older age, lower educational levels, and lower personality dysfunction emerged as significant covariates that were positively associated with higher behavioral compliance.

**Figure 2:**
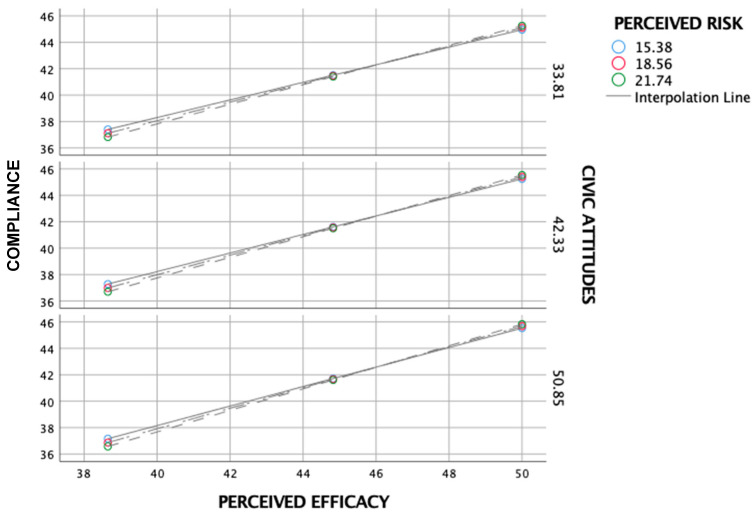
Simple slope analysis of the effect of perceived risk and civic attitudes on the relationship between perceived efficacy and compliance.

As shown in Figure 2, the simple slope analysis found that the positive relationship between perceived efficacy and behavioral compliance was significant (B = 0.78, *p* < 0.001) under both high (+1 SD) and low levels of perceived risk (−1 SD) (B = 0.70; *p* < 0.001). Similarly, the positive relationship between perceived efficacy and behavioral compliance was significant (B = 0.78, *p* < 0.001) under both high and low levels of civic attitudes (B = 0.70, *p* < 0.001). 

### 4.4. Machine Learning

To predict individuals’ compliance with the COVID-19 protective measures based on the collected psychosocial variables (RQ1), participants were split into two classes: high compliance and low compliance. The cut-off for low versus high compliance was set to a total compliance score (i.e., the sum of the scores of all items concerning application of the safety measures) of 30, representing the midpoint of the total compliance score range (i.e., 10–50). Thus, the low compliance class included participants with a total compliance score ≤ 30 (*N* = 171); the high compliance class included participants with a total compliance score > 30 (*N* = 2595). 

We followed the recommended procedure to ensure model generalization and to increase the replicability of the results, by splitting the data into two sets: a training set (used to train and validate the model) and a test set (used to test model accuracy on examples that had never been seen by the ML classifier) [52,60]. In the present study, a percentage split of 80:20 training to test data was applied, with participants randomly assigned to one or the other set while maintaining the proportion between classes. Therefore, the training set consisted of 2213 participants (2076 high compliance and 137 low compliance), and the test set consisted of 553 participants (519 high compliance and 34 low compliance).

All the 14 collected variables were entered in the ML models as predictors: gender, age, education, work position, marital status, citizenship, child(ren), home/work, going out per day, self-efficacy, risk perception, civic attitudes, perceived efficacy, and personality disfunction. Using these 14 predictors, ML algorithms were run and validated on the training sample (*N* = 2213) using a 10-fold cross-validation procedure. Specifically, k-fold cross-validation was used; this resampling procedure seeks to reduce variance in the model performance estimation by using a single training set and a single test set. It portions the sample into k = 10 subsets (folds), using 9 of them to train the model and the remaining subset to validate its accuracy. This procedure is repeated k = 10 times, with 1-fold left out each time as a validation set [61]. The final model metrics are obtained by averaging the metrics of the k = 10 validation subsets. Finally, the models developed through the 10-fold cross-validation were tested on the test sample (*N* = 553). Specific ML algorithms were selected to represent different classification strategies: logistic regression [62], support vector machine [63], naïve Bayes [64], and random forest [65]. This ensured that the results were stable across classifiers and did not depend on specific model assumptions.

In running the classification algorithms, the class imbalance problem was addressed. ML methods work best with balanced datasets—a condition that is rarely met within scientific research with human subjects [66]. A different number of instances across classes may lead to an overall correct classification into the majority class and a complete misclassification of instances into the minority class. In the present research, the two classes were extremely unbalanced, with a ratio of 1:15 between the high compliance and low compliance groups. One strategy to overcome class imbalance consists of altering the relative costs associated with misclassifying the minority and majority classes, in order to compensate for the imbalance [67]. Following this methodology, in the present study, the ML algorithms were designed in such a way that an error in classifying the minority class (low compliance) was weighted 15 times more than an error in classifying the majority class (high compliance). This cost-modifying strategy has been shown to provide better results in addressing class imbalance than other methods, such as random oversampling of the minority class or random undersampling of the majority class [67]. Moreover, it should be noted that, for the goal of this task, it was more beneficial to obtain a lower number of false negatives than false positives; likewise, it was more beneficial to have a model with high sensitivity rather than high specificity. In other words, it was more important to not miss people with low compliance than to misclassify people as high compliance.

The final classification results of the 10-fold cross-validation and test set are reported in Table 5 and Table 6, respectively. The models’ predictive performance was quantified using the following metrics: ROC area, accuracy, precision, recall (or sensitivity), and *F*-measure (F1 score). Of note, the classifiers showed an ROC area in the range of 0.82–0.91 in the test set. However, random forest and naïve Bayes classifiers highlighted a lower recall for low compliance compared to other classifiers, making them weaker models for the purposes of prediction.

Finally, to investigate the weight of the 14 predictors in the models, here we report the point-biserial correlation (r_pb_) between the outcome and each predictor: perceived efficacy = 0.547; self-efficacy = 0.173; age = 0.104; going out per day = 0.101; perceived risk = 0.100; civic attitudes = 0.095; work/home = 0.080; child(ren) = 0.079; education = 0.077; marital status = 0.076; personality dysfunction = 0.072; gender = 0.060; work position = 0.057; citizenship = 0.029.

## 5. Discussion

Public willingness to comply with the protective health measures proposed by authorities is critical for controlling the outcomes of an infectious disease outbreak. The situation in many countries during the current COVID-19 pandemic indicates that, despite legal penalties and mass information campaigns, not all citizens have adopted the recommended behaviors to prevent the spread of the virus. Thus, the present study sought to identify which psychological and psychosocial factors might improve compliance. 

The results confirmed our first research question, showing that self-efficacy significantly mediated the relationship between perceived efficacy and compliance. Stated differently, our findings suggest that individuals who perceive themselves as able to carry out (i.e., those with self-efficacy) those behaviors judged as effective in reducing the threat (i.e., behaviors with perceived efficacy) are more likely to comply with the government measures. This finding highlights the key role of self-efficacy in the adoption and maintenance of recommended health actions [21] and suggests a relationship between self-efficacy and compliance with the preventive measures during the COVID-19 pandemic, as previously reported for different populations (e.g., healthcare workers) [68,69]. Furthermore, some covariates were also significant: older age, lower education levels, and lower levels of personality dysfunction were all associated with increased compliance. These results parallel preliminary findings on the COVID-19 outbreak, indicating that younger persons and those with higher education levels are less likely to comply with the recommended measures, especially those related to hygiene [70]. To the best of our knowledge, this study represents the first attempt to provide data on the association between personality functioning and compliance with health measures. The findings suggest that overall personality functioning may be significant in influencing individuals to adopt the protective measures recommended by authorities. For this reason, personality functioning should be assessed more frequently, and those already known to have a personality impairment (e.g., clinical patients) should be supported and controlled more promptly than others.

Our second and third research questions were only partially confirmed: on the one hand, risk perception and civic attitudes as moderators made the mediation of self-efficacy insignificant, thereby invalidating research questions 2b, 2c, 3b, and 3c. With respect to this unexpected result, we propose that the two relevant moderators made self-efficacy less important in influencing compliance with the health measures; however, further research investigating the associations between these variables is recommended. On the other hand, both risk perception and civic attitudes were found to significantly moderate the relationship between perceived efficacy and compliance. Thus, the impact of perceived efficacy on compliance varied in accordance with risk perception and civic attitudes, thereby confirming research questions 2a and 3a. As the moderation coefficient for both constructs was positive, higher risk perception and civic attitudes were associated with a stronger effect of perceived efficacy on compliance. Regarding risk perception, this result is in line with previous studies reporting a strong association between risk perception and changes in (or maintenance of) health behaviors in a variety of contexts, including epidemic/pandemics [26,27,28,71]. With respect to civic attitudes, this is a novel finding of our study, as such attitudes have not previously been studied in the context of pandemic behavioral responses. The current finding of a link between civic attitudes and preventive health actions suggests the importance, for instance, of teaching civic education in the early school grades. 

Finally, as regards the ML classification models outcome (RQ1), it has been shown that the above-mentioned psychological and psychosocial variables are able to predict which individuals have high versus low compliance, with an ROC area in the range of 0.82–0.91 and high sensitivity for the target class (low compliance). Taken together, the results of the moderated mediation and ML models underline that the most important variable for compliance with the recommended health behaviors is perceived efficacy, as has been consistently indicated by previous studies on behavioral responses to epidemics [15,16,28]. This result suggests that, to foster compliance, communications regarding COVID-19 containment measures should focus on perceived efficacy by highlighting the utility of the recommended behaviors. 

## 6. Conclusions

Overall, our results indicated significantly lower scores in behavioral compliance compared to efficacy perception. The introduction of risk perception and civic attitudes as moderators rendered the mediating effect of self-efficacy insignificant. The impact of perceived efficacy on the adoption of recommended behaviors varied in accordance with risk perception and civic engagement. Finally, the ML classification models’ outcome showed that the psychological and psychosocial variables considered are able to predict which individuals have high versus low compliance.

The present results should be interpreted with caution, due to some limitations. First, the cross-sectional study design, implemented during the first phases of the COVID-19 outbreak in Italy, prevented us from drawing causal inferences. We were unable to assess individuals’ psychological functioning before the virus spread, and we were similarly unable to report behavioral compliance with the recommended health measures in more advanced phases of the outbreak. Furthermore, the data collection via a web-based survey relied on voluntary sampling and self-reported data; thus, the data may be distorted by selection or social desirability biases. Indeed, the survey clearly indicated the health authority recommendations (i.e., “It is suggested that…”). Thus, by disagreeing with the written precautions, participants would have revealed their violation of the official recommendations. Given that such violations would have been seen as socially undesirable, participants may have been reluctant to disclose this. 

Despite these limitations, our findings have several significant implications due to the lack of relevant research on targeted interventions to enhance public compliance with government health recommendations during the COVID-19 outbreak. Government awareness communications and campaigns regarding COVID-19 and related protective measures should be tailored to specific segments of the population, as defined by age and level of education. Furthermore, countries affected by COVID-19 should consider relevant psychological dimensions alongside their lockdown protocols [72]. In particular, government strategists may use the findings of the present study on the psychological characteristics of people who do and do not comply with the containment measures (i.e., perceived efficacy, risk perception, civic attitudes) to target their COVID-19 communications more effectively. Overall, we believe that our findings will be helpful in guiding age-tailored information/advertising campaigns in countries affected by COVID-19 and directing further research on behavioral compliance.

## Figures and Tables

**Table 1:** Descriptive statistics and intercorrelations.

Dimensions	*M*	*SD*	1	2	3	4
1. Compliance	41.66	6.20	-			
2. Perceived efficacy	44.82	6.17	0.742 **	-		
3. Self-efficacy	12.55	1.71	0.332 **	0.198 **	-	
4. Perceived risk	18.56	3.18	0.129 **	0.218 **	−0.077 **	-
5. Civic attitudes	42.33	8.52	0.191 **	0.176 **	0.243 **	0.118 **

Note: ** *p* < 0.01.

**Table 2:** Descriptive statistics and *t*-test results for safety behavior scores.

Safety Measures	Perceived Efficacy *N* = 2766 M (SD)	Compliance *N* = 2766 M (SD)	*t*	*p*	*Cohen’s d*
1. Avoid hugs	3.94 (1.1)	3.76 (1.2)	9.006	<0.001	0.171
2. Avoid handshakes	4.50 (0.8)	4.48 (0.8)	1.253	0.210	0.024
3. Keep one meter away from others	4.42 (0.9)	4.10 (1)	17.653	<0.001	0.336
4. Avoid drinking from bottles and glasses used by others	4.65 (0.6)	4.54 (0.8)	9.978	<0.001	0.190
5. Avoid crowded places	4.67(0.6)	4.56 (0.7)	10.708	<0.001	0.204
6. Disinfect hands at home	4.28 (0.9)	4.09 (1)	12.304	<0.001	0.234
7. Disinfect hands outside	4.63 (0.7)	4.31 (0.9)	22.219	<0.001	0.423
8. Avoid touching face with hands	4.50 (0.8)	3.23 (1.2)	58.744	<0.001	1.117
9. Cough or sneeze into a tissue or elbow	4.63 (0.7)	4.24 (0.9)	26.660	<0.001	0.507
10. Stay at home	4.60 (0.7)	4.34 (0.9)	17.617	<0.001	0.335

**Table 3:** Mediation results (*N* = 2766).

Predictors	β	*t*	*p*	95% CI	
				LL	UL
Model 1 (DV: Self-efficacy)					
Covariates					
Age	−0.00	−0.25	0.800	−0.005	0.004
Education	−0.12	−2.88	0.004	−0.205	−0.039
Personality dysfunction	−0.03	−8.25	<0.001	−0.031	−0.019
Independent variable					
PE	0.05	10.23	<0.001	0.043	0.063
	*R^2^* = 0.06				
	*F*(4, 2761) = 46.69 ***				
Model 2 (DV: Compliance)					
Covariates					
Age	0.04	6.49	<0.001	0.026	0.049
Education	−0.27	−2.68	0.008	−0.467	−0.072
Personality dysfunction	−0.04	−5.75	<0.001	−0.057	−0.028
Independent variables					
SE	0.65	14.38	<0.001	0.561	0.738
PE	0.72	57.44	<0.001	0.691	0.740
	*R^2^* = 0.60				
	*F*(5, 2760) = 826.83 ***				

Notes: DV= Dependent Variable; PE = perceived efficacy of the recommended health measures; SE = self-efficacy. Bootstrap sample size = 5000 (two-tailed); significant values outlined in bold. *** *p* < 0.001.

**Table 4:** Moderated mediation results (*N* = 2766).

Predictors	β	*t*	*p*	95% CI	
				LL	UL
Model 1 (DV: Self-efficacy)					
Covariates					
Age	−0.00	−1.29	0.198	−0.008	0.002
Education	−0.12	−2.89	0.004	−0.201	−0.038
Personality dysfunction	−0.02	−7.45	<0.001	−0.028	−0.016
Independent variables					
PE	0.07	2.50	0.013	0.015	0.126
Risk perception	−0.08	−1.34	0.179	−0.206	−0.038
PE x Risk perception	0.00	0.001	0.846	−0.002	0.003
CE attitudes	0.07	3.15	0.002	0.027	0.117
PE x CE attitudes	−0.00	−1.23	0.217	−0.002	0.000
	*R^2^* = 0.12				
	*F*(8, 2757) = 48.08 ***				
Model 2 (DV: Compliance)					
Covariates					
Age	0.04	6.53	<0.001	0.027	0.050
Education	−0.25	−2.51	0.012	−0.453	−0.056
Personality dysfunction	−0.04	−5.44	<0.001	−0.056	−0.025
Independent variables					
SE	0.52	1.69	0.092	−0.085	1.132
PE	0.34	4.74	<0.001	0.196	0.473
SE x Risk perception	−0.00	−0.14	0.885	−0.029	0.025
SE x CE attitudes	0.01	0.80	0.422	−0.005	0.013
Risk perception	−0.53	−2.52	0.012	−0.952	−0.119
PE x Risk perception	0.01	3.56	<0.001	0.005	0.019
CE attitudes	−0.23	−3.09	0.002	−0.370	−0.083
PE x CE attitudes	0.01	3.34	0.001	0.002	0.007
	*R^2^* = 0.61				
	*F*(11, 2754) = 382.97 ***				

Notes: DV= Dependent Variable; PE = perceived efficacy of the recommended health measures; CE = civic engagement; SE = self-efficacy. Bootstrap sample size = 5000 (two-tailed); significant values outlined in bold. *** *p* < 0.001.

**Table 5:** Metrics of the machine learning (ML) models trained and validated using 10-fold cross-validation.

Algorithm	Accuracy	ROC Area	Class	Precision	Recall	*F*-Measure
Logistic	88.07%	0.941	High compliance	0.990	0.882	0.933
			Low compliance	0.325	0.861	0.904
SVM	88.89%	0.871	High compliance	0.990	0.880	0.932
			Low compliance	0.322	0.861	0.468
Random forest	95.71%	0.938	High compliance	0.976	0.979	0.977
			Low compliance	0.662	0.628	0.644
Naive Bayes	94.35%	0.929	High compliance	0.978	0.916	0.970
			Low compliance	0.534	0.679	0.598

**Table 6:** Metrics of the ML models tested on 553 new participants (test set).

Algorithm	Accuracy	ROC Area	Class	Precision	Recall	*F*-Measure
Logistic	86.62%	0.918	High compliance	0.983	0.873	0.942
			Low compliance	0.283	0.765	0.413
SVM	87.34%	0.823	High compliance	0.983	0.881	0.929
			Low compliance	0.295	0.765	0.426
Random forest	94.39%	0.901	High compliance	0.964	0.977	0.970
			Low compliance	0.556	0.441	0.492
Naive Bayes	94.03%	0.875	High compliance	0.969	0.967	0.968
			Low compliance	0.514	0.529	0.522
